# Comparison of optical myopia control interventions: effect on peripheral image quality and vision

**DOI:** 10.1364/BOE.486555

**Published:** 2023-06-06

**Authors:** Petros Papadogiannis, Charlie Börjeson, Linda Lundström

**Affiliations:** Department of Applied Physics, Royal Institute of Technology (KTH), Stockholm, Sweden

## Abstract

This study compares the effects on peripheral vision and image quality of four myopia control interventions: a) Perifocal spectacles/ArtOptica, b) Stellest spectacles/Essilor), c) MiyoSmart spectacles/Hoya and d) MiSight contact lenses/CooperVision. Five subjects participated with habitual or no correction as reference. Three techniques were used: 1) Hartmann-Shack sensors for wavefront errors, 2) double-pass imaging system for point-spread-functions (PSF), and 3) peripheral acuity evaluation. The results show that multiple evaluation methods are needed to fully quantify the optical effects of these myopia control interventions. Perifocal was found to make the relative peripheral refraction (RPR) more myopic in all subjects and to interact with the natural optical errors of the eye, hence showing larger variations in the effect on peripheral vision. MiSight had a smaller effect on RPR, but large effect on peripheral vision. Stellest and MiyoSmart also showed small effects on RPR but had broader double-pass PSFs for all participants, indicating reduced retinal contrast. Reduction in peripheral retinal contrast might thereby play a role in slowing myopia progression even when the peripheral refraction does not turn more myopic.

## 1. Introduction

The global prevalence of myopia is growing rapidly with increasing risk for vision impairment according to the World Health Organization [1]. Younger age of myopia onset results in longer time for progression and greater risk of developing high myopia in adulthood. This increases the risk of developing sight-threatening conditions like glaucoma [2], cataract [3], retinal detachment [4] and myopic maculopathy [5]. A study has shown that reducing myopia progression by 1 diopter (D) will decrease the likelihood of developing myopic maculopathy by 40% [6]. Therefore, it is of large clinical interest to reduce the axial growth of the eye and the development of myopia. Several different interventions are currently in use for this purpose, but the underlying mechanisms are yet to be understood.

Studies on animal models (monkeys, chickens) have shown that not only the foveal, but also the peripheral optical errors can affect eye growth [7]. Even in the presence of a clear foveal image the eye can still elongate and become myopic when the peripheral image is located behind the retina, so called hyperopic relative peripheral refraction (RPR) [8,9]. Similarly, there is strong evidence that myopic defocus in the periphery can inhibit axial elongation [8,10]. Studies on humans with multifocal contact lenses have shown that center distance designs (more positive power closer to the edge of the pupil) reduced the progression of myopia by -0.73 D in spherical equivalent and 0.32 mm in axial length compared to regular spectacles for myopic children (age 8-12 years old) [11,12], whereas contact lenses with less positive power closer to the pupil edge can increase the axial length in hyperopic children [13]. It is thereby likely that the peripheral retina can detect the sign of the vergence of the light.

Currently, a number of optical myopia control interventions aim to impose defocus on the peripheral retina while central vision is corrected for distance. These myopia control treatments can be in the form of contact lenses (orthokeratology [14–16], soft multifocal contact lenses [11,12]) or spectacles (bifocal spectacles [17], progressive addition spectacles [18,19], spectacles with incorporated lenslets [20–23], diffusion spectacles [24]).

This study concentrates on three spectacle designs for myopia control that have been found to be effective in slowing myopia progression: spectacles with horizontal progressive addition (60% less progression in spherical equivalent compared to single vision spectacles during 4-5 years) [19]; spectacles with highly aspherical lenslets (67% less progression in spherical equivalent compared to single vision spectacles during 2 years) [22]; and spectacles with defocusing lenslets (52% less progression in spherical equivalent compared to single vision spectacles during 2 years) [23]. The treatment principles of these optical interventions are all, at least partly, thought to be related to induced peripheral myopic defocus. However, it has not yet been investigated to what extent they affect the peripheral image quality in the eye and peripheral vision. Therefore, this study compares the effect on peripheral image quality and vision of the three spectacle interventions with the aim to identify common mechanisms for the treatment effect. Additionally, the results are compared to those of an earlier study by Papadogiannis et al. [25] on multifocal contact lenses for myopia control.

## 2. Methods

**Fig. 1.**
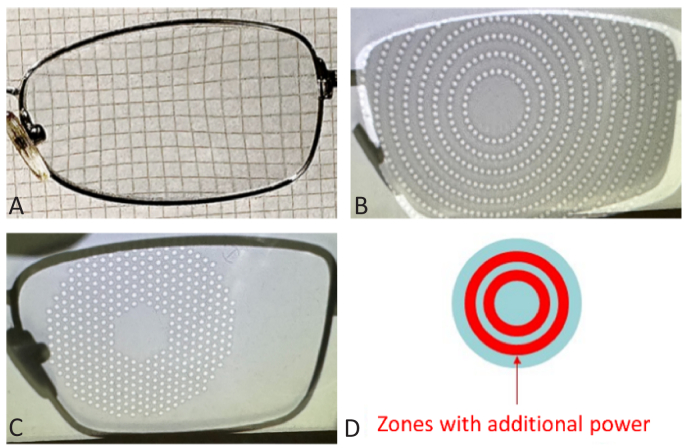
Designs of the four optical myopia control interventions that are reported in this study. A) is the Perifocal lens from ArtOptica, B) is the Stellest lens from Essilor, C) is the MiyoSmart lens from Hoya and D) is the MiSight contact lens from CooperVision.

Three optical myopia control spectacles were investigated in experiments 1 and 2:

a) Perifocal Ms/Ps spectacles from ArtOptica (Fig. 1(A)), which have horizontal progressive addition (up to 2.50 D nasal starting at 4.50 mm from the optic center; and up to 3.00 D temporal starting at 3.00 mm from the optic center) [19].
b) Stellest spectacles from Essilor (Fig. 1(B)), which incorporate defocusing lenslets (1.10 mm in diameter and 3.50 D to 6.00 D in power) in their design in order to achieve addition for myopia control [21,22]. The lenslets are highly aspherical and placed in concentric circles around a clear zone of 10 mm in diameter [26].
c) MiyoSmart spectacles from Hoya (Fig. 1(C)), which have a honeycomb patterned addition area with defocusing lenslets (1.00 mm in diameter and ≈ 4.00 D in power) to control myopia [20,23] and a clear zone of 9 mm [26].

The spectacles were custom-ordered for each of the subjects that participated in the study, to ensure that refraction was correct and matched that of the habitual spectacle correction. Throughout the experiments and before every measurement, the participants verified whether their central visual field was in the clear zone and their peripheral visual field was within the treatment zone of the spectacles. Three different techniques were used for the evaluation of the spectacles: 1) Hartmann-Shack wavefront sensors, 2) double-pass point-spread-function (PSF) imaging system, and 3) peripheral acuity evaluation. The study was approved by the Swedish Ethical Review Authority and conformed to the tenets of the Declaration of Helsinki. After the procedures were explained, written consent was obtained from all participants.

### 2.1 Experiment 1

In the first experiment, peripheral image quality and vision in the 30° nasal visual field were investigated. Five healthy adult subjects were fitted with Perifocal, Stellest, and MiyoSmart spectacles. Subjects 1 (43 years old) and 2 (32 years old) were myopic with tested eye refractions -1.75 DS/-0.5 DC x 140° and -2.75 DS/-0.25 DC x 175° respectively, and subjects 3,4 and 5 (25, 28 and 25 years old respectively) were emmetropic. The subjects were looking straight through the spectacles while the image quality and vision were investigated in the nasal visual field of the right eye. For reference, habitual single vision spectacles were used for the myopes, and no spectacles were used for the emmetropes. The duration of experiment 1 was approximately 2 hours per subject.

#### 2.1.1 Wavefront measurements (technique 1)

Central and peripheral (30° nasal visual field) wavefront errors of the right eye of all subjects were recorded simultaneously for 10 seconds using an experimental dual-angle open-field Hartmann-Shack wavefront sensor (HSWS) [27]. The acquisition rate was 6 Hz. [27]. During measurements, the light in the room was turned off and the subject was fixating binocularly on a Maltese cross situated 3.25 m away from the eye. Three repetitions were made for each tested pair of spectacles, with the light turned on between the repetitions.

The wavefront measurements were reconstructed with Zernike coefficients up to the 7th order. Refractive errors (mean sphere $M=-4\sqrt{3}*C_{2\;}^{0}\;/\;r_{pupil}^{2}$ and astigmatism as Jackson cross-cylinder in 90/180 $J0=-2\sqrt{6}*C_{2}^{2}\;/\;r_{pupil}^{2}$ ) were then calculated from the Zernike coefficients for each acquired wavefront. Relative peripheral refraction (RPR) for natural pupil was acquired by subtracting the foveal refractive error (mean sphere) from the peripheral for coinciding peripheral and foveal wavefronts. Additionally, point-spread-functions (PSFs) were calculated from the Zernike coefficients (scaled to 3.5 mm diameter) for each wavefront for the defocus level corresponding to the 3.25 m distance to the fixation target. Average PSFs were then calculated for each subject and intervention, with the repetitions weighted equally.

#### 2.1.2 Double-pass measurements (technique 2)

In addition to the wavefront measurements, through-focus PSFs were measured with an instrument from Diestia Systems, Athens [28,29]. The instrument images double-pass (DP) PSFs of the eye with different levels of defocus and/or different artificial pupil diameters. For this experiment, the range of imposed defocus was from -11.95 D to +5.95D in steps of 0.1 D, and the pupil diameter was set to 3.5 mm. The same room, illumination, fixation target and distance were used as for the wavefront measurements. PSFs were measured separately for all spectacles for the central and 30° nasal visual field of the right eye of subject 1, 2, and 3, with three repetitions for each measurement. Just as for the wavefront measurements, the subject had an open field-of-view and fixated on the target binocularly. The PSFs for -0.35 D defocus (the value closest to the accommodative demand) were extracted and averaged by averaging the pixel values of three images (one image from each repetition). To ensure that the PSF for the target distance corresponding to -0.35 D defocus was successfully acquired, the through-focus PSFs were analyzed after each measurement, and if the subject blinked around –1 to 0 D of defocus, that measurement was discarded and retaken.

#### 2.1.3 Psychophysical procedure

Peripheral vision was evaluated monocularly with natural pupil size for all optical myopia control interventions. Resolution acuity measurements were performed in the 20° and 30° nasal visual field of the subjects. A Maltese cross was used for fixation and peripheral thresholds were recorded for the left or the right eye of the participants. A chin-forehead-rest mounted on an adjustable mount was used for stability.

An adaptive psychophysics program implemented in MATLAB and Psychophysics toolbox was used for stimuli presentation. The stimulus pattern consisted of Gabor gratings with a standard deviation of 1.6° for the Gaussian window and was presented on a calibrated monitor for 500 ms. The orientation of the gratings was oblique (-45° and 45°) to avoid any neural preferences due to the meridional effect [30]. The subject had to identify the orientation of the gratings in a two-alternative forced choice paradigm by pressing the corresponding key on a keypad; a sound cue was played in the beginning of each trial to ensure that the subject was aware of the stimulus presentation. When the subjects could not resolve the stimulus, they were instructed to guess; guess rate was set to 50% and lapse rate was set to 5%. The spatial frequency of the gratings and the resolution thresholds were determined by Bayesian psychophysical procedures. The subjects were not receiving any feedback whether they were responding correctly or not, and the acuity was determined in 40 trials. All measurements were repeated three times. Measurements with a standard deviation of ≥ 0.1 logMAR were discarded, as were measurements where the Bayesian function failed to converge. A test round was performed to ensure that the procedure was adequately understood by the subjects, and breaks were given as required to avoid fatigue.

##### 2.1.3.1 Peripheral acuity evaluation (technique 3)

Psychophysical measurements were performed on all five subjects for all spectacles. The fixation target was the same Maltese cross used in the objective measurements; this time situated at 2.95 m away from the eye due to room constraints. Resolution cut-off tests at 100% contrast were performed on the right eye at 30° nasal visual field. The left eye was occluded for the psychophysical tests. To avoid fatigue bias, the spectacles were changed between each measurement.

### 2.2 Experiment 2

The effect of the MiSight multifocal contact lenses on low-contrast (10%) peripheral vision was studied earlier by Papadogiannis et al. [25] The MiSight multifocal contact lens has a center-distance design (i.e., central correction for myopia) with +2.00 D treatment zones for myopia control [11,12,31].

The purpose of the second experiment was to repeat the measurements under the same conditions as the aforementioned study for the three myopia control spectacles and compare the results.

Two of the subjects that participated in the study by Papadogiannis et al. [25] were recruited to repeat the experiment in the current study. Subjects 1 and 2 in this experiment correspond to subjects 7 and 4 respectively in the previous study by Papadogiannis et al. Habitual single vision spectacles were used for reference, and the tested myopia control spectacles centrally matched the habitual refraction of the subjects.

Peripheral vision evaluation with low-contrast (10%) grating resolution acuity measurements at 20° nasal visual field were performed monocularly for all spectacles (see details under ‘’ Psychophysical procedure’’ above). The subject was fixating on a Maltese cross mounted 2.6 m away, and thresholds were obtained for the left or right eye while the fellow eye was occluded. The duration of experiment 2 was approximately 1.5 hours per subject.

## 3. Results

### 3.1 Experiment 1

#### 3.1.1 Peripheral refraction (technique 1)

**Fig. 2.**
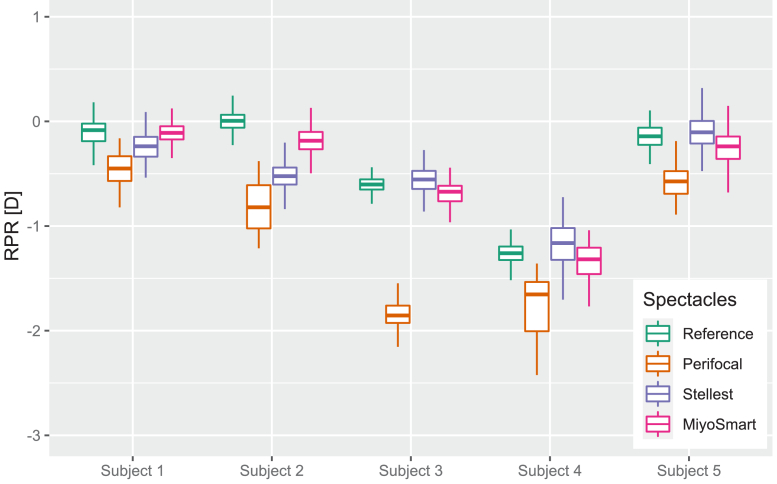
Relative peripheral refraction (RPR) as calculated from simultaneous foveal and peripheral wavefront measurements in the 30° nasal visual field for five subjects with the different myopia control interventions compared to the reference case.

The results of the RPR calculations from the wavefront sensors can be found in Fig. 2. Each box shows median and quartiles for the measured RPR values. Boxes are grouped per subject, with the different interventions indicated by color. It appears that the only intervention that has a consistent effect on RPR is Perifocal, which makes the RPR noticeably more myopic for all subjects. Stellest also displays a myopic shift for subject 2, but not for subjects 1,3,4 and 5 compared to the reference. MiyoSmart does not display a myopic shift in any of the subjects.

**Fig. 3.**
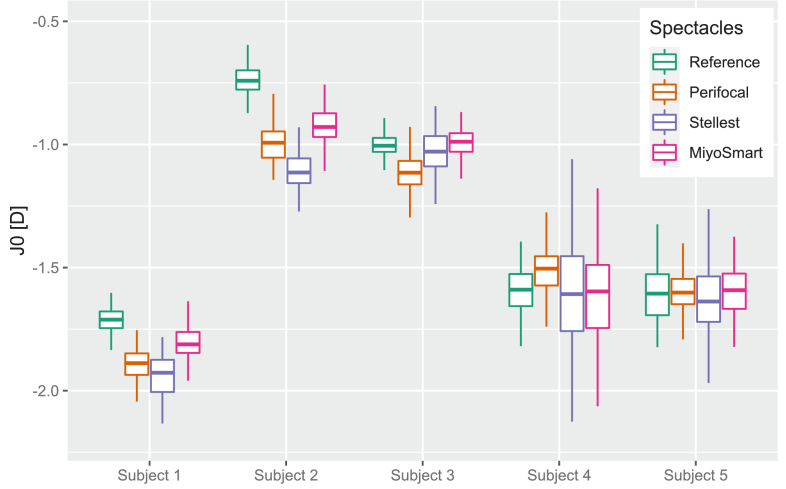
Peripheral astigmatism as Jackson cross-cylinder in 90/180 (J0) calculated from wavefront measurements in the 30° nasal visual field for five subjects with the different myopia control interventions compared to the reference case.

The calculated values of peripheral astigmatism (J0) from the wavefront sensors can be seen in Fig. 3. There is no consistent trend between the different subjects: for the myopic subjects 1 and 2 all myopia control spectacles induce astigmatism, but the effect varies between the interventions; and for the emmetropic subjects 3,4 and 5 the interventions appear to have almost no effect on the astigmatism.

#### 3.1.2 Point-spread functions (techniques 1 and 2)

**Fig. 4.**
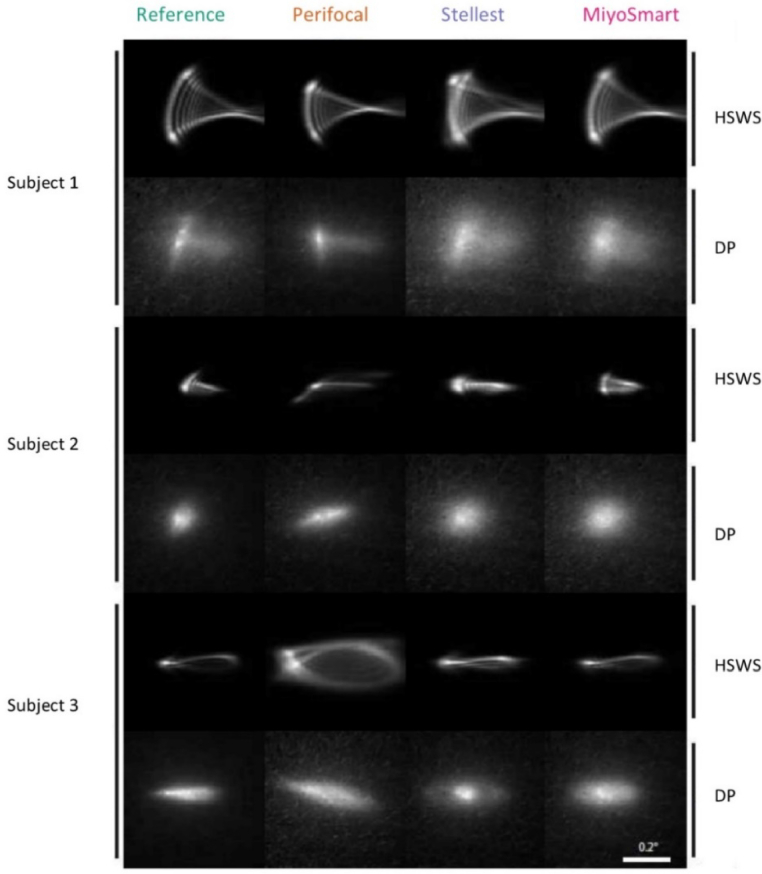
Peripheral point-spread-functions (PSFs) in the 30° nasal visual field for subject 1, 2, and 3 with the different interventions and measurement techniques, measured and calculated for a 3.5 mm pupil diameter. HSWS: Hartmann-Shack wavefront sensor (technique 1); DP: double-pass (technique 2). Each image is an average of three repetitions.

Peripheral PSFs from the two separate techniques in the 30° nasal visual field are shown and compared in Fig. 4 for the different myopia control interventions. The PSFs correspond to the accommodative demand of -0.35 D for a pupil diameter of 3.5 mm. The individual variation in peripheral image quality is large, but there are some consistent trends throughout subjects:

In the double-pass images (DP technique 2), the PSFs are broader for Stellest and MiyoSmart compared to the reference for all subjects. However, this scattering effect is not seen in the wavefront PSFs (HSWS technique 1).

Compared to reference, Perifocal makes the PSF smaller for subject 1, more elongated for subject 2, and much broader for subject 3. These effects are seen both in the wavefront images and double-pass images and are due to Perifocal’s progressive design; the location of the -0.35 D PSF will shift relative to the interval of Sturm depending on the subject’s baseline RPR and astigmatism.

**Fig. 5.**
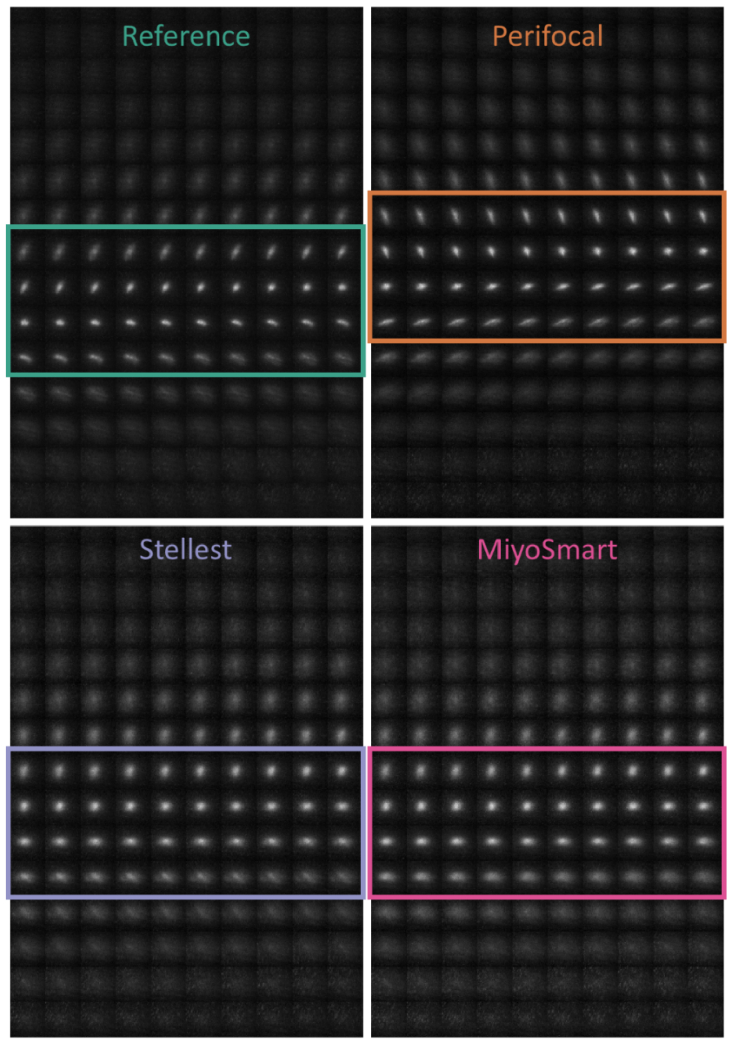
Peripheral through-focus double-pass point-spread-functions (PSFs) in the 30° nasal visual field for subject 2 during one repetition with each intervention, measured for a 3.5 mm pupil diameter. The defocus steps range from -7.95 D in the upper left corner of each panel to +5.95 D in the lower right. The change in defocus between each PSF is 0.1 D, each row therefore corresponds to 1.0 D change in defocus. The colored squares inscribe the 4.0 D region with best PSFs, which were located between -1.95 D and +1.95 D with the reference, Stellest, and MiyoSmart, but between -2.95 D and +0.95 D with Perifocal.

The through-focus PSFs from the double-pass instrument agree with the previous results in that a shift towards more myopic RPR compared to the reference is only seen with Perifocal. This is consistent for all three subjects and Fig. 5 shows one example from the through-focus measurements on subject 2. It can also be seen that the image quality of the best PSF is reduced compared to the reference for Stellest and MiyoSmart, whereas Perifocal produces a best PSF that is similar to the reference albeit shifted in position relative to the retina.

#### 3.1.3 Peripheral acuity evaluation (technique 3)

**Fig. 6.**
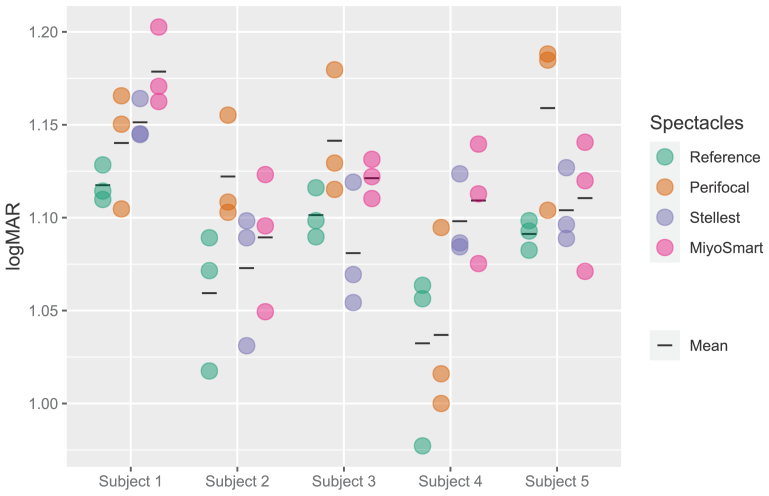
Peripheral high contrast grating resolution acuity in the 30° nasal visual field for the five subjects and the different myopia control interventions. Circles indicate repetitions, black lines indicate means of the three repetitions. Lower values correspond to better acuity.

Data from the 100% contrast psychophysical measurements can be found in Fig. 6 and the differences from the reference case in logMAR (intervention threshold minus reference thresholds) are presented in Table 1. As can be expected for peripheral high contrast resolution, the effect of the different intervention designs is small and similar to the variation between repetitions. Still, some tendencies can be seen. For instance, Perifocal worsens resolution noticeably in subjects 2, 3 and 5, and to some extent in subject 1. The effect of Stellest is less consistent and only gives a clear worsening for subjects 1 and 4. MiyoSmart worsens the resolution in all subjects, most noticeably in subjects 1 and 4.

**Table 1.** Differences in mean peripheral high contrast grating resolution acuity.^*a*^

	Subject 1	Subject 2	Subject 3	Subject 4	Subject 5
**Perifocal**	0.023	0.063	0.040	0.005	0.068
**Stellest**	0.034	0.013	-0.020	0.066	0.013
**MiyoSmart**	0.061	0.030	0.020	0.077	0.019

^*a*^ Differences in mean peripheral high contrast grating resolution acuity in logMAR in the 30° nasal visual field for the myopia control interventions compared to the reference case (intervention threshold minus reference threshold, positive values indicating worse vision with intervention).

### 3.2 Experiment 2

The effects on peripheral vision of the three spectacles for myopia control (Perifocal, Stellest and MiyoSmart) were evaluated and compared to those of MiSight contact lenses and single-vision spectacles [25].

**Fig. 7.**
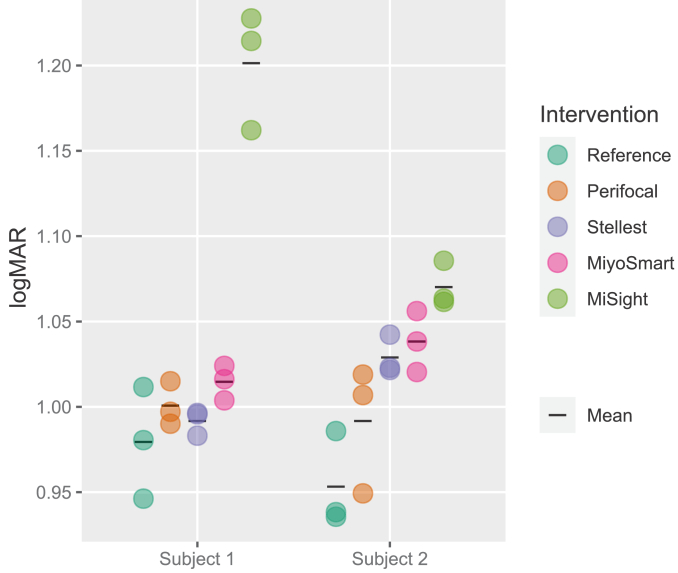
Peripheral low contrast (10%) grating resolution acuity in the 20° nasal visual field for the two subjects and the different myopia control interventions. Circles indicate repetitions, black lines indicate means of the three repetitions. Lower values correspond to better acuity.

Figure 7 presents the low-contrast (10%) peripheral resolution thresholds in the 20° nasal visual field of subject 1 and 2 in logMAR, and Table 2 presents the logMAR differences from the reference case (intervention threshold minus reference thresholds). In both subjects the MiSight lens reduced the peripheral contrast the most, followed by MiyoSmart. Additionally, variability in the effects on contrast within the subjects can be observed.

**Table 2.** Differences in mean peripheral low contrast (10%) grating resolution acuity.^*a*^

	Subject 1	Subject 2
**Perifocal**	0.021	0.038
**Stellest**	0.012	0.076
**MiyoSmart**	0.035	0.085
**MiSight**	0.222	0.117

^*a*^ Differences in mean peripheral low contrast (10%) grating resolution acuity in logMAR in the 20° nasal visual field for the myopia control interventions compared to the reference case (intervention threshold minus reference threshold, positive values indicating worse vision with intervention).

## 4. Discussion

The aim of this study was to identify possible treatment mechanisms in the off-axis performance of four optical myopia control interventions: spectacles with horizontal progressive addition (Perifocal); spectacles with highly aspherical lenslets (Stellest); spectacles with defocusing lenslets (MiyoSmart); and multifocal contact lenses (MiSight). To tackle the challenges posed by the large optical errors of the peripheral eye together with the myopia control interventions, in-depth measurements were performed utilizing wavefront aberrations (measured by a Hartmann-Shack sensor), through-focus double-pass point-spread-functions (obtained from a double-pass imaging system), and peripheral acuity evaluation (using psychophysical methods) on a few subjects fitted with all four different designs. In short, the results show that none of the interventions improve the peripheral image quality compared to habitual standard spectacle correction and that the shift towards a more myopic peripheral refraction, if any, is small.

All four interventions incorporate treatment zones with more positive power than the distance correction, which can be expected to induce peripheral myopic defocus. However, RPR is difficult to measure both because the location of the best peripheral image is poorly defined due to the large depth-of-focus and because it requires simultaneous control of the central refractive state. Therefore, the current study incorporates wavefront sensing simultaneously for the peripheral and the central visual field, as well as through-focus double-pass measurements. Both measurement techniques showed that only Perifocal with progressive addition produced a clear shift of around 1 D to a more myopic peripheral refraction compared to the reference case for all three subjects. The other three designs produced small (see Fig. 2 of this study) or inconsistent (see Table 3 in the study from Papadogiannis et al. [25]) shifts in RPR. It should, however, be noted that all four designs induced or preserved myopic RPR (except for one of the eight subjects in the earlier study with MiSight multifocals [25]), thereby still offering a peripheral vergence cue that might function as a treatment mechanism.

The fact that the primary optical effect of Stellest, MiyoSmart and MiSight is not a more myopic RPR, despite the positively powered treatment zones, can be understood as an effect of the large variation in optical power over the pupil in peripheral angles. This multifocality with multiple overlapping images induces more high-order optical errors and therefore a more evenly smeared out PSF with lower contrast already for low spatial frequencies. This is interesting since recent studies show that myopic subjects are not very responsive to positive defocus and that contrast reductions can be a possible treatment effect of myopia control interventions [24,32,33]. The contrast reduction in the image on the peripheral retina is clear both from the double-pass and the peripheral acuity evaluations. The largest effect on peripheral vision, and hence the largest reduction in contrast, that was found in the current study was produced by the MiSight contact lenses, followed by MiyoSmart.

The reduction in contrast for the spectacles with lenslets (Stellest and MiyoSmart) is more difficult to assess with the wavefront sensor compared to double-pass and vision evaluations. This might be because the scattered light from the lenslets is not registered by the wavefront sensor, since we do not have enough resolution for measuring as high order scattering as the double-pass system. Similarly, modal wavefront reconstruction and analyses of individual Zernike polynomials is less efficient for such highly irregular wavefronts. For example, the Zernike coefficients for off-axis astigmatism shows a large increase with all four myopia control interventions when assessed with the wavefront sensor for subject 1, which cannot be seen in the computed PSF nor in the through-focus double-pass measurements. However, the double-pass technique also pose challenges in that the light passes twice through the eye, which amplifies scattering and reduces asymmetric features in the PSF (even though the pupil size for the incoming and the outgoing light is not the same [34]). Peripheral acuity evaluation is powerful in that a clear reduction in vision can be directly related to a reduction in retinal image contrast, but it is also depending on the pupil size and can be influenced by the level of neural adaptation. We have therefore limited our discussion here to optical effects that are seen by more than one of our evaluation methods.

We have not been able to find studies from other authors that have measured the image quality on the peripheral retina through these optical myopia control interventions. However, theoretical and optical bench testing studies agree with our results in that the best peripheral image becomes less well-defined with the studied interventions [35–37]. For example, the recent study from Gantes-Nuñez et al. shows how the non-coaxial design of Stellest and MiyoSmart acts to maintain the best image quality in the focal plane of the base optic (designed for distance correction) in spite of the positively powered lenslets, thereby not inducing any myopic RPR compared to single vision spectacles [26]. It should also be noted that the peripheral optical effects investigated in the current study are not the only possible treatment effects by the optical myopia control interventions. For example, there are indications that accommodation can be affected by the interventions [38].

The optical design of a myopia control intervention interacts with the optical properties of the natural eye, which leads to individual variations in the image quality on the retina. This can also be seen in the results of this study and may explain some of the variation in treatment effect between children. The optical outcome of some designs, such as those of Perifocal and MiSight, depend on and interact more with the optical errors of the individual eye than the spectacle designs with lenslets. One hypothesis is that when the more subject-depending designs match with the individual eye they offer more effective treatment by alternating the RPR, whereas the lenslet-designs produce a lower but less subject-dependent treatment mainly through contrast reduction. To verify or reject this hypothesis, longitudinal studies on children with peripheral optical evaluations through the myopia control intervention are needed.

## Data Availability

Data underlying the results presented in this paper are not publicly available at this time but may be obtained from the authors upon reasonable request.
